# The complete mitochondrial genomes of two endangered bitterling *Acheilognathus tabira tohokuensis* and *A. tabira erythropterus* (Cyprinidae, Acheilognathinae)

**DOI:** 10.1080/23802359.2019.1661304

**Published:** 2019-09-03

**Authors:** Nobuaki Nagata, Jyun-Ichi Kitamura

**Affiliations:** a Division of Collections Conservation, National Museum of Nature and Science, Ibaraki, Japan;; b Mie Prefectural Museum, Mie, Japan

**Keywords:** Complete mitogenome, *Acheilognathus tabira*, bitterling, subspecies

## Abstract

*Acheilognathus tabira* (tabira bitterling) comprises of 5 subspecies, all of which are endangered. In this study, the mitochondrial genome (mitogenome) of the 2 subspecies, *A. tabira tohokuensis* and *A. tabira erythropterus,* whose mitogenomes have not been reported previously, was determined. The total lengths of *A. tabira tohokuensis* and *A. tabira erythropterus* mitogenomes were 16,774 bp and 16,770 bp, respectively, and were noted as slightly AT-rich. Phylogenetic analysis revealed that these 2 subspecies of *A. tabira* were the most closely related, out of the 5 subspecies. The deciphered mitogenomes would be useful for conservation and evolutionary studies.

The bitterling is a freshwater fish that lays eggs on the gill chambers of living mussels and has diversified in Asia, including Japan. However, almost all species of Japanese bitterling species are at a risk of extinction due to the deteriorating river environment and the invasion of predatory alien fish such as largemouth bass, *Micropterus salmoides* and the competitive alien bitterling, *Rhodeus ocellatus ocellatus* and *Acheilognathus macropterus*. The phylogeny and phylogeography of the Japanese bitterling is relatively well studied (Miyake et al. 2011; Kitamura et al. 2012; Chang et al. 2014; Saitoh et al. 2016; Nagata et al. 2018). Recently, environmental DNA has been described useful in bitterling conservation studies (Sakata et al. 2017), and mitochondrial information has become important. *Acheilognathus tabira* is endemic to Japan and has 5 subspecies (Arai et al. 2007). This species is listed as NT (near threatened) in the IUCN red list (Miyazaki et al. 2019), whereas, Ministry of the Environment, Government of Japan; lists all 5 subspecies as IA (critically endangered,) or IB (endangered). *Acheilognathus tabira tohokuensis* and *A. tabira erythropterus* are distributed in the Japan Sea side and the Pacific Ocean side of eastern Honshu, Japan, respectively. Both are listed as IB in the Japanese Red Data List, however, the total length of mitochondrial DNA has not been reported.

*Acheilognathus tabira tohokuensis* and *A. tabira erythropterus* were collected from Niigata prefecture (37.91 N, 139.31 E) and Ibaraki prefecture (35.95 N, 140.52 E), Japan, respectively. A part of the pelvic fin was collected and stored in 99% ethanol, following which the collected individuals were immediately released as they were endangered species. Total DNA were extracted using Wizard^®^ DNA purification kit (Promega, Madison, WI). The vouchered DNA has been deposited in the National museum of Nature and Science, Japan (NSMT-DNA67767 for *A. tabira tohokuensis*, NSMT-DNA67768 for *A. tabira erythropterus*). The whole mitogenome was amplified into two fragments by the PCR. Following which, the fragments were sequenced using Miseq sequencer (illumina, San Diego, CA). The total reads were assembled using NOVOPlasty (Dierckxsens et al. 2017) and the mitogenome was annotated using Mitoanotator in MitoFish webserver (Iwasaki et al. 2013).

Length of the entire mitogenome of *A. tabira tohokuensis* (GenBank/DDBJ/EMBL accession number LC494269) and *A. tabira erythropterus* (LC494270) was 16774 bp and 16770 bp, respectively. Both of them were observed to possess 2 ribosomal RNA genes, 22 transfer RNA genes, 13 protein-coding genes (PCGs), and a control region (D-loop). The genomes of both the subspecies were slightly AT-rich, with a ratio of 56%. Maximum likelihood phylogenetic analysis based on 13 PCGs using RAxML (Stamatakis 2014) revealed that the 5 subspecies were monophyletic (Figure 1). In addition, *A. tabira erythropterus* and *A. tabira tohokuensis* were most closely related, and this observation was consistent with the previous studies (Kitamura et al. 2012). The genetic distances of *Cytb* and D-loop (only with apparent total length) among the subspecies were 3.24–8.15% and 3.05–3.39%, respectively. These mitogenomes would be useful for conservation and evolutionary studies through intraspecific phylogeny and environmental DNA.

**Figure 1.**
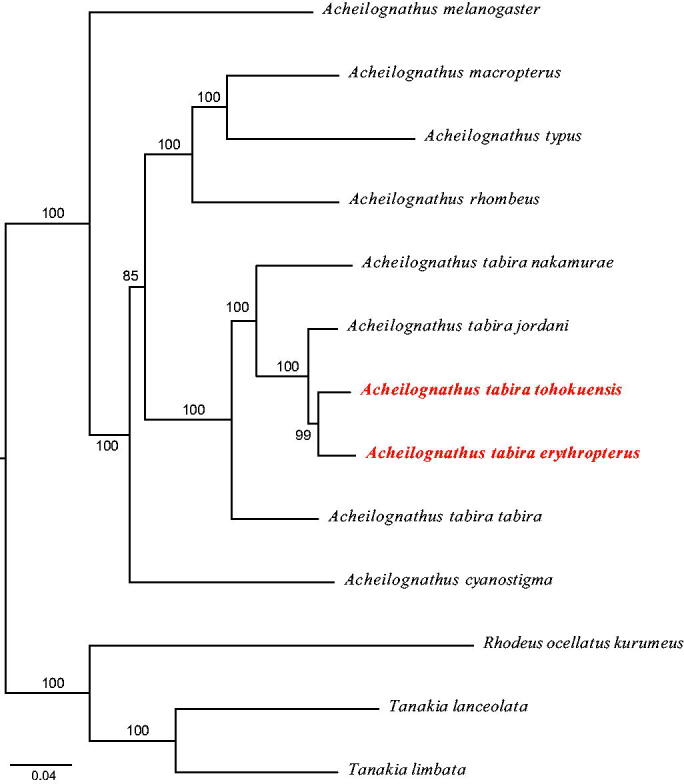
Maximum likelihood phylogenetic tree of 13 bitterlings based on 13 protein coding genes: *Acheilognathus rhombeus* (AP013342) (Miya & Sado unpublished), *Acheilognathus macropterus* (EF483935) (Hwang et al. 2014), *Acheilognathus tabira tohokuensis* (LC494269), *Acheilognathus tabira erythropterus* (LC494270) (this study), *Acheilognathus tabira tabira* (AP013344), *Acheilognathus tabira jordani* (AP013343), *Acheilognathus tabira nakamurae* (AP013347), *Acheilognathus cyanostigma* (AP013346) (Miya unpublished), *Acheilognathus melanogaster* (AP012985) (Saitoh et al. unpublished), *Acheilognathus typus* (AB239602), *Rhodeus ocellatus kurumeus* (AB070205) (Saitoh et al. 2006), *Tanakia lanceolata* (KJ589418) (Xu et al. 2016), *Tanakia limbata* (KM386633) (Luo et al. 2016). The number beside each node indicate bootstrap values in percentage based on 1,000 replications.
